# New County Medical Societies

**Published:** 1891-08

**Authors:** C. W. LeGrand, Van B. Thornton

**Affiliations:** President; Secretary


Still Organizing.—Stimulated by the advocacy of the JOURNAL the profession of Texas is still organizing for the better advancement of science, and for influence to secure the enactment of much needed legislation. Dr. J. W. Cartwright, of Amarillo, writes :

"The physicians of the Panhandle proper met to-day (August 10) in my office, for the purpose of organizing a medical society. Will send you particulars in due time."

And Dr. Van B. Thornton, of Hempstead, writes, July 25:

"On the 23rd of July, the following physicians of Hempstead and Waller county met at Dr. LeGrand's office in Hempstead, for the purpose of organizing a medical society, to be known as the Waller County Medical Association : Drs. Levi Mahan, J. H. Morrison, C. W. LeGrand, Van B. Thornton, of Hempstead, and Dr. L. L. Mahan, of Sunny Side. Dr. LeGrand was elected president ; Dr. L. L. Mahan, vice-president, Dr. Van B. Thornton, secretary ; Drs. J. H. Morrison, Levi Mahan and L. L. Mahan were appointed a committee to enlist the physicians of the county to join with us on the 1st Monday in August, at 11:00 o'clock in the permanent organization.

*Resolved*, That DANIEL'S TEXAS MEDICAL JOURNAL, of Austin, be notified by the secretary and asked to give notice of the same. C. W. LEGRAND, President.

VAN B. THORNTON, Secretary.

